# Prevention of cervical cancer in HIV-seropositive women from developing countries through cervical cancer screening: a systematic review

**DOI:** 10.1186/s13643-018-0874-7

**Published:** 2018-11-17

**Authors:** Witness Mapanga, Brendan Girdler-Brown, Shingairai A. Feresu, Tsungai Chipato, Elvira Singh

**Affiliations:** 1 School of Health Systems and Public Health, Epidemiology & Biostatistics, University of Pretoria, 5-10 H.W. Snyman Building, Pretoria, South Africa; 2 Faculty of Health Sciences, University of Fort Hare, 45 Church Street, Gasson Building, 7th Floor, P.O. Box 1054, East London, 5201 South Africa; 3 Department of Obstetrics and Gynaecology, College of Health Sciences, University of Zimbabwe, Avondale, Harare Zimbabwe; 4 Cancer Epidemiology Research Group, National Cancer Registry, National Health Laboratory Service, Johannesburg, South Africa; 5 Harare, Zimbabwe

**Keywords:** Cervical cancer, Prevention, HPV, HIV, Developing countries

## Abstract

**Background:**

There is scanty or inconclusive evidence on which cervical cancer screening tool is effective and suitable for human immunodeficiency virus (HIV)-seropositive women. The aim of this review was to assess, synthesise and document published evidence relating to the available cervical cancer screening modalities for HIV-seropositive women in developing countries. This paper did not review the issue of human papillomavirus (HPV) prophylactic vaccine on HIV-seropositive women.

**Methods:**

Five electronic databases were systematically searched from inception to January 2018 for relevant published original research examining cervical cancer prevention modalities for HPV infection, abnormal cytology and direct visualisation of the cervix amongst HIV-seropositive women in developing countries. Extra studies were identified through reference list and citation tracking.

**Results:**

Due to methodological and clinical heterogeneity, a narrative synthesis was presented. Of the 2559 articles, 149 underwent full-text screening and 25 were included in the review. Included studies were of moderate quality, and no exclusions were made based on quality or bias. There is no standard cervical cancer screening test or programme for HIV-seropositive women and countries screening according to available resources and expertise. The screening methods used for HIV-seropositive women are the same for HIV-negative women, with varying clinical performance and accuracy. The main cervical cancer screening methods described for HIV-seropositive women are HPV deoxyribonucleic acid/messenger RNA (DNA/mRNA) testing (*n* = 16, 64.0%), visual inspection with acetic acid (VIA) (*n* = 13, 52.0%) and Pap smear (*n* = 11, 44.0%). HPV testing has a better accuracy/efficiency than other methods with a sensitivity of 80.0–97.0% and specificity of 51.0–78.0%. Sequential screening using VIA or visual inspection with Lugol’s iodine (VILI) and HPV testing has shown better clinical performance in screening HIV-seropositive women.

**Conclusion:**

Although cervical cancer screening exists in almost all developing countries, what is missing is both opportunistic and systematic organised population-based screenings. Cervical cancer screening programmes need to be integrated into already existing HIV services to enable early detection and treatment. There is a need to offer opportunistic and coordinated screening programmes that are provider-initiated to promote early identification of cervical precancerous lesions.

**Systematic review registration:**

PROSPERO CRD42018095702

**Electronic supplementary material:**

The online version of this article (10.1186/s13643-018-0874-7) contains supplementary material, which is available to authorized users.

## Introduction

With the increase in cervical cancer morbidity and mortality in developing countries, concern has shifted to how much can be done to prevent this public health challenge especially in those who are immunocompromised. With the advent of HIV in most of these developing countries especially those in sub-Saharan Africa, the burden, incidence and mortality due to cervical cancer are increasing [1–4]. HIV/AIDS in most developing countries has resulted in high cervical cancer incidence, and because of this, cervical cancer has been classified as an AIDS-defining disease [2–4]. HIV-seropositive women have been found to be at higher risk of HPV infection due to their immune-compromised status and that they are 2 to 12 times more likely to develop cervical precancerous lesions that lead to cervical cancer than HIV-negative women [2, 3]. Globally, 1 to 2% of HIV-negative women develop CIN stages 2 and 3 annually whilst HIV-positive women are at 10% more prone to developing CIN stages 2 and 3 [5]. In addition, HIV is itself associated with other multiple enabling factors for cervical cancer such as lower economic status, multiple sexual partners, early sexual debut and smoking [6], and this makes prevention of cervical cancer in HIV very important.

Cervical cancer prevention is important in reducing morbidity and mortality, and there are a number of screening methods available. The screening methods include cytological-based tests (Pap smear/glass slide cytology and liquid-based cytology), HPV DNA testing and visual inspection tests (with acetic acid (VIA) and with Lugol’s iodine (VILI)) [7–9]. Pap smear has been used widely in developed countries for many years with success [10, 11]. In developing countries, the lack of proper infrastructure and qualified personnel to carry out the procedure and interpret the results has hampered the utilisation of Pap smear [12, 13].

However, regardless of the available screening methods and considerable evidence in reducing the burden of cervical cancer, epidemiological and health system challenges and constraints still exist in most developing countries that make it difficult for some cervical cancer screening strategies and initiatives to be available. In addition, there is a lack of both opportunistic and organised systematic population-based screenings amongst HIV-seropositive women due to fewer resources, resulting in uncoordinated screening with any available screening method. With the introduction of mass HPV vaccination for young girls in some developing countries, opportunities to offer the vaccine to HIV-positive middle-aged women through HIV health programmes exist. The safety and immunogenicity of HPV vaccine are almost comparable in HIV-positive and HIV-negative women [14, 15]. Offering HPV vaccination, as primary cervical cancer prevention to HIV-positive women, will reduce cervical cancer incidence and morbidity.

Despite a number of evidence-based guidelines, strategies and research on cervical cancer screening or prevention in low-resource settings, slow progress in the implementation of these guidelines due to the lack of implementation experts has become a public health challenge that requires urgent solutions to mitigate the morbidity and mortality due to cervical cancer. There is little rigorous synthesised evidence on which cervical cancer screening methods are being used for HIV-seropositive women, if these current screening methods are the same for HIV-negative women and if these screening methods are effective for HIV-seropositive women. The review aims to answer the following questions: What are the screening modalities that are used to prevent cervical cancer in HIV-seropositive women in developing countries? Are these the same screening modalities that are used for HIV-negative women? Are the screening modalities effective in HIV-seropositive women? This review will investigate, identify and synthesise the existing screening modalities being used for cervical cancer in HIV-seropositive women in developing countries. Synthesising research from developing countries can provide robust evidence of context-specific cervical cancer screening methods for HIV-seropositive women to fill the gap in evidence.

## Methods

### Search strategy

**Fig. 1:**
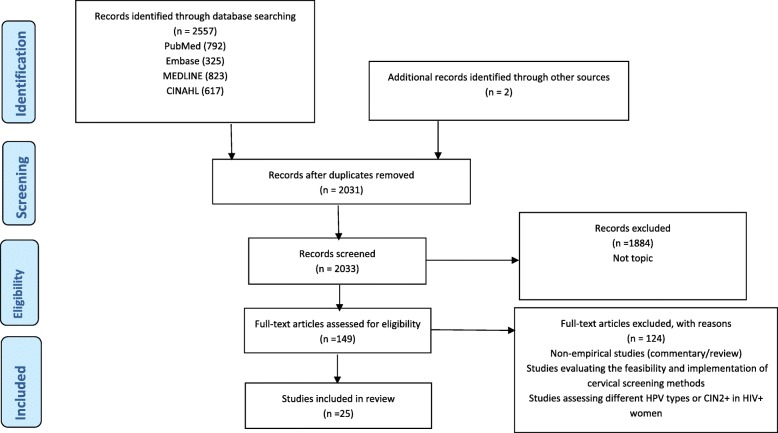
Search strategy. The search strategy is reported according to the PRISMA guidelines

This systematic review was registered with PROSPERO (CRD42018095702) and was carried out guided by a protocol (PROSPERO CRD42017054678) [16] and reported according to the PRISMA guidelines (Fig. 1) [17].

Two independent reviewers (WM and TC) guided by a protocol [14] searched PubMed (via the PubMed/MEDLINE interface using the “PICO” option), CINAHL (via the EBSCO interface using keywords), Cochrane (via The Cochrane Library using MeSH terms and qualifiers), Embase and MEDLINE (via the OvidSP interface) using keywords and supplementary free-text terms until January 2018. The search terms (cervical cancer or cervical neoplasms or cervical carcinomas) AND (prevention or screening or Pap smear or VIA) AND (developing countries or underdeveloped countries or low-income countries) were used specifically for each database (an additional PDF file shows this in more detail [see Additional file 1]).

### Eligibility criteria

Articles were eligible for inclusion based on inclusion criteria: original research that assessed or reported cervical cancer prevention methods for HIV-positive women (Pap smear, VIA, HPV DNA testing), peer-reviewed, reported in English and done in or for regions or countries which are considered developing by the United Nations [16]. Reviews, commentaries and studies assessing cervical cancer in general were excluded from the review.

### Study selection

The initial search of the databases yielded 2557 results, and additional two studies were identified through citation and reference tracking to make a combined 2559 articles. Two independent reviewers (WM and SF) conducted the screening process to identify eligible studies, and reasons for excluding studies were documented (see Fig. 1). Disagreements and other issues related to the screening process were resolved as reported in the protocol [16]. Removal of duplicates and screening of title and abstracts excluded 2212 articles. An additional 198 articles were further excluded because of not being relevant to the topic. The remaining 149 articles were reviewed in full text, and 124 studies were excluded for not meeting the eligibility criteria (an additional PDF file shows these excluded studies in more detail [see Additional file 2]). Twenty-five articles met the eligibility criteria and were included for final analysis.

### Data extraction

The primary reviewer (WM) and TC double extracted the following content from the included 25 studies: title of the study, author, publication year, study design, study setting (country/region), sample size, exposures and outcomes and all results including statistics. Three additional team members, SF, BGB, and ES, assessed the extracted data to ensure accuracy, and inconsistencies were discussed and resolved through consensus. Frequency tables were used to summarise the results.

### Quality assessment of included studies

The quality of the included studies was assessed using a combination of a modified version of the Newcastle-Ottawa Quality Assessment Scale and the NIH Study Quality Assessment Tools for observational cohort cross-sectional case-control and before-after studies [18, 19]. The focus of research, key findings, study design, length of follow-up and representativeness of participants were used to ascertain quality. For an easy quality assessment process, studies were categorised into three groups namely randomised controlled trials, observational studies with control group(s) and observational studies without control group(s) [16]. Outcome measures were assessed based on whether the articles had a predefined outcome measure and if any cervical cancer prevention method was explored or its application was discussed [16]. Two independent reviewers (WM and BGB) carried out the quality assessment process, and discrepancies that arose were resolved through discussion with other team members. The average score of the two reviewers (WM and BGB) on both the quality assessment tools became the quality score for each study, with 0 being very low quality and 5 being high quality.

## Results

**Fig. 2:**
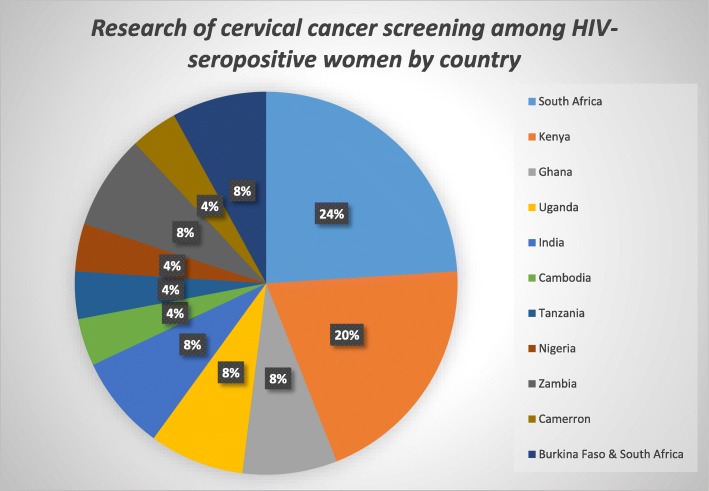
Research of cervical cancer screening amongst HIV-seropositive women by country

Out of 2559 articles, 25 met the inclusion criteria and were synthesised for results. Table 1 summarises the study characteristics and evidence extracted from the studies respectively. Twenty-two studies (88.0%) were conducted in and for sub-Saharan Africa, 2 (8.0%) in Asia and 1 (4.0%) in South America (Fig. 2). All the included studies were published within the last decade, 2008 to 2018.

**Table 1:** Table of evidence

First author, year	Purpose	Population and age	Country	Prevention method/tool	Study type	Outcome(s)	Results	Authors’ conclusions	Quality score
Bansil et al., 2015	To evaluate and compare the performance of 3 cervical cancer screening options amongst HIV-infected women in Uganda	2337 HIV-positive and HIV-negative women; 25 and 60 years	Uganda	-Visual inspection with acetic acid (VIA) -Vaginal careHPV -Cervical careHPV	Observational study without a control group	Clinical performance of cervical careHPV, vaginal careHPV and VIA	Amongst HIV-positive women, cervical careHPV had sensitivity and specificity of 94.3% and 62.4%, respectively; vaginal careHPV had 80.0% sensitivity and 59.9% specificity; VIA had 77.1% sensitivity and 47.3% specificity. Amongst HIV-negative women, VIA had 93.8% sensitivity and 60.5% specificity; cervical careHPV had 81.3% sensitivity and 80.9% specificity; vaginal careHPV had 75.0% sensitivity and 81.9% specificity.	CareHPV™ performs better for screening cervical cancer amongst HIV-positive women when compared to VIA. VIA can be used as a triage method and reduce the number of treatment by half	Moderate
Cholli et al., 2018	Assess the feasibility and clinical outcomes of screening HIV-positive and HIV-negative Cameroonian women by pairing VIA/VILI-DC with careHPV (high-risk HPV nucleic acid test)	913 previously unscreened HIV-positive and HIV-negative women; ≥ 30 years	Cameroon	(VIA/VILI-DC) careHPV	Observational study without a control group	VIA/VILI-DC and careHPV co-testing strength amongst HIV-positive and HIV-negative women	For HIV-positive women, 8% (29/384) tested VIA/VILI-DC positive whilst 41% (157/384) tested positive on careHPV test (*p* < 0.0001). HIV-positive women had more than twice VIA/VILI-DC positive results (*n* = 29/384, 7.6%) than HIV-negative women (*n* = 15/530, 2.8%) (*p* < 0.0001). HIV-positive women were almost twice (1.9 times) more likely to test careHPV positive than HIV-negative for each VIA/VILI-DC result category.	Exclusively relying only on VIA/VILI-DC will likely result in about 50% overtreatment rate. Due to their weaknesses, pairing VIA/VILI-DC and HPV DNA testing is effective.	Moderate
Chung et al., 2013	To compare Papanicolau smear, visual inspection with acetic acid and human papillomavirus cervical cancer screening methods amongst HIV-positive women by immune status and antiretroviral therapy	500 HIV-positive women; 18 and 55 years	Kenya	Pap smear VIA HPV test colposcopy-directed biopsy	Observational study without a control group	Sensitivity, specificity of Pap smear, VIA and HPV testing	Individually, the most sensitive test was Pap (ASCUS+) (92.7%), which was significantly more sensitive than VIA (62.7%; *p* < 0.001), Pap (HSIL+) (71.8%; *p* < 0.001) and HPV (83.6%; *p* = 0.04) (Table 3). HPV was significantly more sensitive than VIA (*p* < 0.001) and Pap (HSIL+) (*p* = 0.04). Pap (HSIL+) (97.1%) was significantly more specific than VIA (65.9%; *p* < 0.001) and HPV (55.7%; *p* < 0.001), and VIA were more specific than HPV (*p* = 0.006). The cervical screening method with the highest AUC was Pap (HSIL+) (0.85), which was significantly greater than VIA (0.64; *p* < 0.001), HPV (0.70; *p* < 0.001), Pap (ASCUS+) (0.71; *p* < 0.001) and Pap (LSIL+) (0.76; *p* < 0.001). Combining cervical screening methods did not significantly improve test sensitivity over using Pap (ASCUS+) alone. However, combining VIA and Pap (HSIL+) to confirm positive test results had greater specificity than Pap (HSIL+) alone (99.1 vs. 97.1%; *p* < 0.001).	Pap smear is a robust test amongst HIV-positive women regardless of immune status or ART duration. Pap (ASCUS+) had the highest sensitivity, the combination of both Pap (HSIL+) and VIA positive had the highest specificity and Pap (HSIL+) had the highest AUC.	Moderate
Dartell et al., 2014	To examine the ability of VIA and HPV-testing to detect cytologically diagnosed high-grade lesions or cancer (HSIL+)	3603 HIV-positive and HIV-negative women; 24.4% were 29 years or younger, 35.1% were 30–39 years, 25.2% were 40–49 years and 15.3% were 50 years or older	Tanzania	Conventional cytology VIA HPV-DNA detection	Observational study without a control group	Sensitivity, specificity, positive predictive value and negative predictive value of VIA, HR HPV-testing compared to cytology	Amongst all women, VIA had a sensitivity of 28.5% (95% CI 20.9–36.0) and a specificity of 96.5% (95% CI 95.9–97.1). The sensitivity for VIA was higher in women from urban areas (39.0%) and amongst HIV-positive women (50.0%). HPV-testing had a high sensitivity (94.2%; 95% CI 90.2–98.1) and a somewhat lower specificity (82.8%; 95% CI 81.6–84.1). The specificity was lowest amongst HIV-positive women (58.2%) and amongst women 29 years or younger (74.7%). The VIA and HPV testing had a PPV ranging from 16.7 to 32.6% and from 7.2 to 22.9%, respectively. For both VIA and HPV-testing, the lowest PPV was seen amongst women below 29 years old. NPV was high for both VIA and HPV-testing (> 99.6%) and reached up to 100% for HPV testing amongst women who were below 29 years and amongst women who were HIV positive.	HPV testing would be a better primary screening tool for cervical cancer in sub-Saharan Africa, possibly with VIA as a secondary tool to increase specificity.	Moderate
Firnhaber et al., 2016	To compare VIA, cytology and HPV-DNA testing amongst HIV-positive women	688 HIV-positive women; age IQR (33–44)	South Africa	Pap smear VIA HPV testing	Observational study without a control group		Progression to CIN2+ was higher in women with positive VIA results (12.6%; 24/191) than those VIA-negative (4.4%; 19/432). HPV-positive women at baseline were more likely to progress to CIN2+ (12.3%; 36/293) than those HPV-negative (2.1%; 7/329). Cytology-positive women at baseline were more likely to progress to CIN2+ (9.6%; 37/384) than cytology-negative women (2.5%; 6/237). Approximately 10% (10.4%; 39/376) of women with CIN1 at baseline progressed to CIN2+. Women who were VIA or HPV positive at baseline were more likely to progress aIRR 1.85, (95% CI 1.46 to 2.36), aIRR 1.41 (95% CI 1.14 to 1.75), respectively.	Progression to CIN2+ in HIV-infected women is significant when measured by baseline positive VIA, HPV or Pap, and yearly screening by any method should be considered in this population if possible.	Moderate
Huchko et al., 2014	To determine the optimal strategy for cervical cancer screening in women with HIV infection by comparing two strategies: VIA and VIA followed by VILI in women with a positive VIA result	3462 HIV-positive women; 23–60 years	Kenya	VIA and VIA/VILI	Observational study with a control group	Positivity rate and PPV for VIA and VIA/VILI for CIN2+ amongst HIV-infected women	Screening positivity rate was 26.4% for VIA and 21.7% VIA/VILI (*p* = 0.003) with a follow-up colposcopy rate of 96.6% and 96.3%, respectively. The PPV of VIA for biopsy-confirmed CIN2+ in a single round of screening was 35.2% VIA, compared with 38.2% for VIA/VILI (*p* = 0.41).	The absence of much differences between VIA and VIA/VILI in detection rates or PPV for CIN2+ suggests that VIA can be used alone as a cervical cancer screening strategy in low-income settings.	High
Huchko et al., 2015	To compare the diagnostic accuracy of VIA to VILI for cervical cancer screening in HIV-infected women	654 HIV-positive women; 23–65 years	Kenya	VIA and VILI	Randomised clinical trial	Test performance of VIA or VILI	The test positivity rates were 26.2% for VIA and 30.6% for VILI (*p* = 0.22). The rate of detection of CIN2+ was 7.7% in the VIA arm and 11.5% in the VILI arm (*p* = 0.10). Sensitivity and specificity were 84.0% and 78.6%, respectively, for VIA and 84.2% and 76.4% for VILI. The positive and negative predictive values were 24.7% and 98.3% for VIA, and 31.7% and 97.4% for VILI. Amongst women with CD4+ count < 350, VILI had a significantly decreased specificity (66.2%) compared to VIA in the same group (83.9%, *p* = 0.02) and compared to VILI performed amongst women with CD4+ count ≥ 350 (79.7%, *p* = 0.02).	VIA and VILI had similar diagnostic accuracy and rates of CIN2+ detection amongst HIV-infected women.	High
Joshi et al., 2013	To evaluate an accurate, affordable, and feasible method to screen and treat HIV-infected women so that cervical cancer can be prevented amongst them	1128 HIV-positive women; 21–60 years	India	VIA, VILI, cytology, HPV testing, colposcopy	Observational study without a control group	Concurrent performance of cytology, HPV testing, VIA and VILI in detecting CIN2 and 3	The sensitivity, specificity and positive predictive values for VIA to detect CIN2 and 3 lesions were 83.6, 88.8 and 27.7%, respectively; the corresponding values for VILI were 89.1, 89.3 and 30.1%; for cytology at ASCUS threshold were 63.3, 94.5 and 35.2%; and for HPV testing were 94.6, 77.4 and 17.8%, respectively. Although VIA had a higher sensitivity than cytology, it did not reach statistical significance. HPV testing was 100% sensitive in detecting CIN3 lesions; however, it had significantly lower specificity than VIA, VILI and cytology (*p* < 0.001).	HPV testing, VILI and VIA have a higher sensitivity in detecting high-grade CIN than that of conventional cytology. Sequential testing with VIA and VILI is the most feasible screening approach for cervical cancer screening in HIV-infected women in low-resource countries. When HPV testing becomes feasible and affordable, HPV testing followed by VIA/VILI may be considered.	Moderate
Kuhn et al., 2010	To evaluate the efficacy amongst HIV-infected women of a simpler, screen-and-treat strategy in which all women with a positive screening test are treated with cryotherapy	6555 women, whom 956 were HIV-positive; 35–65 years	South Africa	HPV DNA based screen-and-treat and VIA-based screen-and-treat	Randomised clinical trial	Safety and efficacy of screen-and-treat amongst HIV-positive women	HPV DNA testing was highly effective in reducing the risk of CIN2+ by 36 months amongst both HIV-positive [relative risk (RR) = 0.20, 95% confidence interval (CI) 0.06–0.69] and HIV-negative (RR = 0.31, 95% CI 0.20–0.50) women. The benefit of VIA-and-treat was less marked and only reached statistical significance in HIV-positive women (RR = 0.51, 95% CI 0.29–0.89) and not in HIV-negative women (RR = 0.76, 95% CI 0.52–1.1). The sensitivity of HPV DNA testing at enrolment to detect CIN2+ through 36 months was 94.4% in HIV-positive women, whereas the sensitivity of the VIA test was 63.9% in HIV-positive women. In the HPV and-treat group, there was a slightly lower rate of CIN2+ after cryotherapy amongst HIV-positive (2.8%) vs. HIV-negative (7.1%) women, but this difference was of borderline significance (*p* = 0.05). In the VIA-and-treat group, CIN2+ failure rates after cryotherapy were similar in HIV-positive (4.8%) and HIV-negative (2.8%) women	HPV-based screen-and-treat is safe and effective in HIV-positive women. A single round of screening with an HPV test followed by cryotherapy of all screen-positive women reduced high-grade cervical cancer precursors (CIN2+) by 80%, and this was sustained through 36 months. VIA-based screen-and-treat was significantly less effective, although better than no intervention in HIV-positive women.	High
Lim et al., 2011	To compare Pap smear readings to VIA findings amongst HIV-infected women in Phnom Penh, Cambodia	293 HIV-infected women	Cambodia	Pap smear and VIA	Observational study without a control group	Degree of correlation between Pap smear and VIA findings	55 (19%) women screened positive on VIA; 25 (8.5%) women screened positive by Pap. Visual inspection with acetic acid detected 18 of the 25 patients with abnormal cytology and was normal in 7 women with abnormal cytology. 37 (67%) women with positive VIA were negative by cytology.	Our study shows a reasonable correlation between VIA and Pap smear, with VIA detecting more abnormalities than cytology. In the absence of Pap smear availability, VIA may be a reasonable cervical cancer screening method for HIV-infected women in Cambodia.	Low
Mabeya et al., 2012	To determine the accuracy of VIA vs. Pap smear amongst HIV-infected women	150 HIV-infected women; 20–45 years	Kenya	Pap smear and VIA	Observational study without a control group	Accuracy of VIA vs. conventional Pap smear as a screening tool for CIN/cancer amongst HIV-infected women with biopsy as the reference criterion standard	Using AUC as an overall measure of screening accuracies and using CIN1 or higher as the gold standard threshold, the performance of Pap smear is slightly better than VIA, but the difference is not significant (Pap smear: AUC = 0.596, VIA: AUC = 0.571, *p* = 0.64). When using CIN2 or higher as the gold standard threshold, the performance of Pap smear and VIA are more comparable (Pap smear: AUC = 0.606, VIA: AUC = 0.603, *p* = 0.93). Using CIN2 or higher disease on biopsy as an end point, VIA has a sensitivity of 69.6% (95% CI = 55.1–81.0%), specificity of 51.0% (95% CI = 41.5–60.4%), PPV of 38.6% (95% CI = 28.8–49.3%), and NPV of 79.1% (95% CI = 67.8–87.2%). For conventional Pap smear, sensitivity was 52.5% (95% CI = 42.1–71.5%), specificity was 66.3% (95% CI = 52.0–71.2%), PPV was 39.7% (95% CI = 27.6–51.8%) and NPV was 76.8% (95% CI = 67.0–85.6%).	Visual inspection with acetic acid is comparable to Pap smear and acceptable for screening HIV-infected women in resource-limited settings such as Western Kenya.	Moderate
Michelow et al., 2016	To evaluate the performance of the Cellslide® automated liquid-based cytology (LBC) system as a possible alternative to conventional cytology amongst HIV-positive women	348 HIV-positive women;18–65 years	South Africa	Cellslide® automated LBC	Observational study without a control group	Number of positive and negative samples tested using Cellslide®	For HSIL, Cellslide® showed sensitivity of 76.0% (95% CI 64.8–85.1) and specificity of 91.0% (95% CI 87.0–94.2), with a false-omission rate < 7%, compared with conventional cytology. When compared with conventional cytology, Cellslide® showed sensitivity of 89.6% (95% CI 82.9–94.4) and specificity of 92.2% (95% CI 87.8–95.4) for NILM, sensitivity of 70.2% (95% CI 61.3–78.0) and specificity of 87.7% (95% CI 82.6–91.7) for LSIL and sensitivity of 100% (95% CI 2.5–100) and specificity of 98.8% (95% CI 97.1–99.7) for ASCH.	The performance of the Cellslide((R)) LBC system was similar to that of conventional cytology in this population of high-risk HIV-positive women, indicating that it may be introduced successfully as part of a cervical cancer screening programme.	Low
Mutyaba et al., 2010	To evaluate the ‘see-see and treat’ strategy and the role of HIV on cervical cancer prevention in Uganda	5105 HIV-negative and HIV-positive women; 20–60 years	Uganda	VIA/VILI and cryotherapy	Observational study without a control group	Detection rates by age-group and cervical lesion treatment	Detection rates per 1000 women screened were higher amongst the older women (41–60 years) compared to women aged 20–40 years. They were accordingly 55% and 20% for inflammation, 10% and 2% for LGSIL, 5% and 2% for HGSIL and 6% and 1% for invasive cervical cancer. Of the 608 women, 103 (16%) were HIV positive. HIV positivity was associated with a higher likelihood of inflammation (RR = 1.7; 95% CI 1.2–2.4). The 32 women with SIL (19 LGSIL and 13 HGSIL) underwent treatment by cryotherapy (31 women) or LEEP (1 woman). 1 woman had persistent LSIL and 1 had inflammation; both were HIV positive. Other 27 women had normal findings.	VIA/VILI used as a sole method for cervical cancer screening would entail significant false-positive results. HIV seropositivity was associated with a higher prevalence of inflammatory cervical lesions. Cryotherapy treatment outcome was not conclusive due to the limited follow-up time	Low
Ngou et al., 2015	To compare the Hybrid Capture 2 HPV DNA assay (HC2) and the INNO-LiPA HPV Genotyping Extra assay (INNO-LiPA) for cervical cancer screening in HIV-1-infected African women	1224 HIV-positive women in Burkina Faso (*N* = 604) and South Africa (*N* = 620); 25–50 years	Burkina Faso and South Africa	HC2 and INNO-LiPA	Observational study without a control group	Agreement between HC2 and INNO-LiPA for detection of HR-HPV infection and compare their performances in diagnosing cervical lesions detected by cytology and histology	When considering the 13 h-HPV types detected by HC2, 634 (51.8%) and 849 (69.4%) samples were positive by HC2 and INNO-LiPA, respectively. Agreement between assays was 73.9% [adjusted kappa coefficient value, 0.44 (95% confidence interval 0.43 to 0.53)]. Agreement improved with analysis restricted to women with high-grade cervical lesions [adjusted kappa coefficient value, 0.83 (95% confidence interval 0.74 to 0.91)]. The prevalence of hr-HPV, as determined by HC2 and INNO-LiPA, was 34.5% and 54.5%, respectively, in samples with normal cytology, 48.0% and 68.0%, respectively, in samples with atypical squamous cells of undetermined significance, 51.8% and 75.2%, respectively, in samples with low-grade SIL and 86.3% and 89.8%, respectively, in samples with high-grade SIL/atypical squamous cells that cannot exclude HSIL. Sensitivity, specificity and positive and negative predictive values for the diagnosis of histological high-grade lesions (CIN2+) were 88.8%, 55.2%, 24.7% and 96.7%, and 92.5%, 35.1%, 19.1% and 96.6% for HC2 and INNO-LiPA, respectively	HC2 has lower analytical sensitivity but higher specificity than INNO-LiPA for diagnosing high-grade lesions; the 2 tests presented a comparable clinical sensitivity. HC2 might be suitable for cervical cancer screening in HIV-1-infected African women, but its use in resource-limited settings merits to be further evaluated in comparison with other prevention strategies.	Moderate
Ngou et al., 2013	To compare careHPV and hybrid capture 2 assays for detection of high-risk human papillomavirus DNA in cervical samples from HIV-1-infected African women	149 HIV-1-infected African women (75 in Johannesburg, South Africa and 74 in Ouagadougou, Burkina Faso); 25–50 years	Burkina Faso and South Africa	careHPV and HC2	Observational study without a control group	Agreement in detecting HR-HPV between careHPV and HC2	The HR-HPV DNA detection rates were 37.6% and 34.9% for careHPV and HC2, respectively. Agreement between the two tests was 94.6% (95% confidence interval [CI], 89.7 to 97.7%) with a kappa value of 0.88 (95% CI, 0.81 to 0.96), indicating an excellent agreement.	careHPV may be considered as suitable as HC2 for cervical cancer screening amongst HIV-infected African women.	Moderate
Obiri-Yeboah et al., 2017	To compare the performance of careHPV with HPV genotyping for the detection of cytological cervical squamous intraepithelial lesions (SIL)	175 women (94 HIV-1-seropositive and 81 HIV-seronegative women); ≥ 18 years	Ghana	HPV Genotyping vs. careHPV	Observational study with a control group	Agreement in detecting HR-HPV between careHPV and HPV genotyping	The inter-assay concordance was 94.3% (95% CI 89.7–97.2%, kappa = 0.88), similar by HIV serostatus. The careHPV assay was equally sensitive amongst HIV-1-seropositive and HIV-1-seronegative women (97.3% vs. 95.7%, *p* = 0.50) and slightly more specific amongst HIV-seronegative women (85.0% vs. 93.1%, *p* = 0.10). careHPV had good sensitivity (87.5%) but low specificity (52.1%) for the detection of low SIL or greater lesions, but its performance was superior to genotyping (87.5 and 38.8%, respectively). Reproducibility of careHPV, tested on 97 samples by the same individual, was 82.5% (95% CI 73.4–89.4%).	The performance characteristics of careHPV compared to genotyping suggest that this simpler and cheaper HPV detection assay could offer a suitable alternative for HPV screening in Ghana.	High
Obiri-Yeboah et al., 2017	To determine the acceptability, feasibility and performance of alternative self-collected vaginal samples for HPV detection using careHPV amongst Ghanaian women	194 women (97 HIV-positive); ≥ 18 years	Ghana	Self-collected vaginal samples with care HPV	Observational study with a control group	Performance of self-collected cervico-vaginal samples compared to clinician-collected samples	Overall HPV detection concordance was 94.2% (95% CI 89.9–97.1), kappa value of 0.88 (*p* < 0.0001), showing excellent agreement. This agreement was similar between HIV-positive (93.8%) and HIV-negative (94.7%) women. Sensitivity and specificity of SC compared to CC were 92.6% (95% CI 85.3–97.0) and 95.9% (95% CI 89.8–98.8), respectively. The highest sensitivity was amongst HIV-positive women (95.7%, 95% CI 88.0–99.1) and highest specificity amongst HIV-negative women (98.6%, 95% CI 92.4–100). Overall, 76.3% of women found SC very easy/easy to obtain, 57.7% preferred SC to CC and 61.9% felt SC would increase their likelihood to access cervical cancer screening.	The feasibility, acceptability and performance of SC using careHPV support the use of this alternative form of HPV screening amongst Ghanaian women. This could be a potential new affordable strategy to improve uptake of the national cervical cancer screening programme.	Moderate
Sahasrabuddhe et al., 2012	To rigorously evaluate the clinical accuracy of VIA and cytology amongst HIV-infected women in Pune, India	303 non-pregnant HIV-infected women; 25–40 years	India	VIA Pap smear	Observational study without a control group	Sensitivity, specificity, PPV and NPV for VIA and cytology	At CIN2+ disease threshold, the sensitivity, specificity and positive and negative predictive value estimates of VIA were 80, 82.6, 47.6 and 95.4%, respectively, compared to 60.5, 59.6, 22.4 and 88.7% for the atypical squamous cells of undetermined significance or severe (ASCUS+) cutoff on cytology; 60.5, 64.6, 24.8 and 89.4% for the low-grade squamous intraepithelial cells or severe (LSIL+) cutoff on cytology; and 20.9, 96.0, 50.0 and 86.3% for high-grade squamous intraepithelial lesion or severe (HSIL+) cutoff on cytology. A similar pattern of results was found for women with the presence of carcinogenic HPV-positive CIN2+ disease, as well as for women with CD4+ cell counts < 200 and < 350 muL(−1).	Overall, VIA performed better than cytology in this study with biologically rigorous endpoints and without verification bias, suggesting that VIA is a practical and useful alternative or adjunctive screening test for HIV-infected women.	Moderate
Wu et al., 2016	To measure the sensitivity, specificity and predictive values of p16INK4a ELISA for CIN2+	1054 HIV-infected women; ≥ 23 years	Kenya	p16(INK4a) ELISA	Observational study without a control group	Sensitivity, specificity and predictive values of p16INK4a ELISA	The p16INK4a cutoff value with the highest combined sensitivity (89.0%) and specificity (22.9%) for biopsy-proven CIN2+ was 9 U/mL. The positive predictive value was 13.6% and negative predictive value was 93.8%. Overall, the p16INK4a positivity with the selected 9 U/mL cutoff level was 828 (78.6%) women; in comparison, biopsy-proven CIN2+ was found in only 127 (12%) women.	p16(INK4a) ELISA did not perform well as a screening test for CIN2+ detection amongst HIV-infected women due to low specificity. Our study contributes to the ongoing search for a more specific alternative to HPV testing for CIN2+ detection.	Low
Akinwuntwan et al., 2008	To assess the correlation between cytology and VIA in HIV-positive women	205 consenting HIV-seropositive women; 17–60 years	Nigeria	Pap smear VIA	Observational study without a control group	Sensitivity, specificity, PPV, NPV and diagnostic accuracy of Pap smear and VIA	The sensitivity of VIA was 76.0% (95% CI 52.0–91.0), specificity 83.0% (95% CI 77.0–88.0) and positive predictive value 34.0% (95% CI 21.0–49.0). The sensitivity of Pap smear was 57.0% (95% CI 34.0–77.0), specificity of 95.0% (95% CI 90.0–97.0) and positive predictive value of 55.0% (95% CI 33.0–75.0). Diagnostic accuracy of VIA is 82.0% (95% CI 76.0–87.0) and for Pap smear is 91.0% (95% CI 86.0–98.0)	In HIV-seropositive women, the sensitivity of VIA is 76.0%, making it a useful screening test for pre-invasive lesion of the cervix in low resource settings but not a diagnostic tool.	Moderate
Firnhaber et al., 2013	To compare the sensitivity and specificity of conventional Pap smear screening to that of HPV DNA and VIA testing for detection of histologically confirmed high-grade CIN2+ in HIV-infected women	1202 HIV-infected women; 18–65 years	South Africa	Pap smear, VIA and HPV DNA test using HC2	Observational study without a control group	Sensitivity and specificity of Pap smear, HPV DNA test and VIA	VIA and HPV were positive in 45% and 61% of women, respectively. Estimated sensitivity/specificity for HPV, Pap smear and VIA for CIN2+ was 92%/51.4%, 75.8%/83.4% and 65.4/68.5% (nurse reading), respectively. Sensitivities were similar, and specificities appeared significantly lower for the HPV test, cytology and VIA amongst women with CD4 counts ≤ 200 cells/mm^3^ as compared to CD4 counts > 350 cells/mm^3^.	Although HPV was the most sensitive screening method for detecting CIN2+, it was less specific than conventional cytology and VIA with digital imaging review. Screening programmes may need to be individualised in the context of the resources and capacity in each area.	Moderate
Chibwesha et al., 20165	To determine the clinical performance of VIA, digital cervicography (DC), Xpert HPV and OncoE6 for cervical cancer screening in an HIV-infected population	200 HIV-infected women; ≥ 18 years	Zambia	VIA, DC, Xpert HPV and OncoE6	Observational study without a control group	Sensitivity and specificity of VIA, DC, Xpert HPV and OncoE6	Of the 200 women, 15% were screen positive by VIA, 20% by DC, 47% by Xpert HPV and 6% by OncoE6. Using a CIN2+ threshold, the sensitivity and specificity of VIA were 48% (95% confidence interval [CI] 30–67%) and 92% (95% CI 86–95%), respectively. Similarly, the sensitivity and specificity of DC were 59% (95% CI 41–76%) and 88% (95% CI 82–93%), respectively. The sensitivity and specificity of Xpert HPV were 88% (95% CI 71–97%) and 60% (95% CI 52–68%), respectively. Finally, the sensitivity and specificity of OncoE6 were 31% (95% CI 16–50%) and 99% (95% CI 97–100%), respectively.	VIA and DC displayed moderate sensitivity and high specificity. Xpert HPV performed equivalently to currently approved HPV DNA tests, with high sensitivity and moderate specificity. OncoE6 displayed excellent specificity but low sensitivity. These results confirm an important role for VIA, DC and Xpert HPV in screen-and-treat cervical cancer prevention in low- and middle-income countries, such as Zambia	Low
Adamson et al., 2015	To (1) compare the test positivity between the two collection methods, (2) assess the accuracy and agreement of self-collected tampons compared to clinician-collected specimens for hrHPV mRNA testing and (3) assess the acceptability of the self-collected tampon method	325 HIV-infected women; ≥ 25 years	South Africa	HrHPV messenger RNA (mRNA) test	Observational study without a control group	Sensitivity and specificity of hrHPV mRNA test	Over 90% of women reported no difficulties self-collecting specimens, and 82% were willing to perform the tampon collection at home. Based on clinician collection specimens, the prevalence of hrHPV mRNA in our study population was 36.7% (95% CI 31.4–42.0%). There was no difference in test positivity between clinician collection, 36.7%, and tampon collection, 43.5% (*p* = 0.08). Using clinician collection as the reference test, the sensitivity and specificity for hrHPV mRNA of tampon collection were 77.4% (95% CI 69.8–85.0%) and 77.8% (95% CI 71.9–83.6%), respectively.	Tampon-based self-collection is acceptable to women and has similar hrHPV mRNA positivity rates as clinician collection but has reduced sensitivity and specificity compared to clinician collection.	Moderate
Segondy et al., 2016	To evaluate the performance of careHPV for detecting CIN2+ amongst women living with HIV-1 in Burkina Faso and South Africa	1052 HIV-1-seropositive women; 25–50 years	South Africa and Burkina Faso	careHPV assay INNO-LiPA	Observational study without a control group	Sensitivity, specificity, positive and negative predictive values of careHPV assay	Overall, 45.1% of women had a positive careHPV test (46.5% in BF, 43.8% in SA). The careHPV positivity rate increased with the grade of cytological lesions. Sensitivity and specificity of careHPV for the diagnosis of CIN2+ (*n* = 60, both countries combined) were 93.3% (95% confidence interval (CI) 83.8–98.2) and 57.9% (95% CI 54.5–61.2), respectively. Specificity increased with CD4 count. careHPV had a similar clinical sensitivity but higher specificity than the INNO-LiPA assay for detection of CIN2+.	Results suggest that careHPV testing is a reliable tool for cervical cancer screening in HIV-1-infected women in sub-Saharan Africa.	Moderate
Bateman et al., 2014	To assess the clinical performance of DC, as well as cytology in HIV-infected women	303 women; 20–45 years	Zambia	DC Pap smear	Observational study without a control group	Clinical performance of each screening test to detect cervical lesions on histopathology	The sensitivity of DC for identifying CIN2+ was 84% (95% CI 72–91%), and the specificity was 58% (95% CI 52–64%) (Table 2). The sensitivity estimates of cytology for identifying CIN2+ were as follows: HSIL+, 61% (95% CI 48–72%); LSIL+, 90% (95% CI 80–95%); and ASC-US+, 100% (95% CI 94–100%). The specificity estimates of cytology for identifying CIN2+ were as follows: HSIL+, 58% (95% CI 52–64%); LSIL+, 35% (95% CI 29–41%); and ASC-US+, 13% (95% CI 10–18%). The PPVs were low (23–33%) for both tests, whilst the NPVs were correspondingly high (86–100%). A similar pattern of results was observed at the CIN3+ diagnostic threshold on histopathology (Table 2).	Digital cervicography appears to be as good as cytology in HIV-infected women.	Moderate

All the included studies explored the clinical performance of cervical cancer screening methods/tools on HIV-seropositive women, with a few comparing them to screening HIV-negative women. There was almost a complete consistency in defining the key outcomes across the studies to indicate clinical performance, which is looking at sensitivity, specificity and positive and negative predictive values. However, the baseline characteristics of the study participants including age varied across the studies. In addition, sampling and recruitment of participants, screening process (opportunistic vs. organised), the interval on which follow-ups were conducted and the type of visits (one-visit schemes vs. return visit scheme) were also different. Completeness of data, data management methods, adjustment for confounders and analysis also differed across the studies. Therefore, because of this heterogeneity, a narrative descriptive synthesis was performed.

### Study design

The included studies ranged from cross-sectional to randomised clinical trials. Classifying them according to the protocol [16], most of the included studies were observational studies without control groups (*n* = 20, 80.0%). There were three observational studies with a control group (12.0%) and two (8.0%) randomised clinical trials (see Table 1).

Of the two randomised clinical trials (see Table 1), one compared the diagnostic accuracy between VIA and VILI [20], whilst the other clinical trial evaluated the efficacy and safety of the screen-and-treat using either HPV DNA test or VIA [21]. The three observational studies with a control group compared VIA to the sequential use of VIA and VILI [22], and the other two assessed the performance of careHPV® (a rapid batch diagnostic test for detection of high-risk HPV DNA vs. HPV genotyping [23, 24].

The 20 observational studies without a control group evaluated clinical performance of VIA, careHPV®, VILI with digital cervicography, Pap smear, HPV test, HPV DNA, cryotherapy and Cellslide® automated liquid-based cytology [25–37]. The other studies evaluated the see-see and treat strategy of VIA/VILI and cryotherapy [38], Hybrid Capture-2® (HC2), INNO-LiPA®, p16INK4a ELISA®, Xpert HPV®, high-risk HPV messenger RNA and OncoE6® for HPV detection [39–44].

### Cervical cancer screening methods/tools for HIV-seropositive women

Most of the studies were conducted in sub-Saharan Africa, and they evaluated and compared the performance of VIA, detecting high-risk HPV DNA using careHPV®, INNO-LiPA®, HC2®, Xpert HPV or OncoE6®; a combination of VIA/VILI with digital cervicography; Pap smear; colposcopy and test and treat using VIA/VILI; or HPV DNA and cryotherapy [20–29, 32–44]. The two studies conducted in Asia evaluated VIA, VILI, cytology, HPV testing and colposcopy to find an accurate, feasible and affordable cervical screening method for HIV-infected women [30, 34]. In Cambodia, they compared VIA and Pap smear, looking at the correlation between the two amongst HIV-infected women [31].

### Primary prevention methods

The p16INK4a ELISA®, a surrogate marker for high-risk HPV, was assessed as a potential primary cervical cancer screening tool for HIV-seropositive women in Kenya [41].

### Secondary prevention methods

For secondary prevention, VIA was the most frequently used and evaluated screening method for HIV-seropositive women in 16 of the 25 articles included (*n* = 16, 64.0%). Comparison between Pap smear and VIA to assess which is the better screening method was explored in 4 of the included articles (*n* = 4, 16.0%). Evaluation of Pap smear, VIA, HPV test and colposcopy was also examined in 4 of the studies (*n* = 4, 16.0%), whilst VIA and VILI were assessed in only 1 study (*n* = 1, 4.0%). HPV DNA/mRNA testing with various methods and tools such as HC2®, INNO_LiPA®, HPV genotyping, careHPV®, hrHPV mRNA, Xpert HPV® and OncoE6® was evaluated in 9 studies (*n* = 9, 36.0%). In the test/screen and treat initiatives, HPV DNA and cryotherapy, VIA and cryotherapy, and VIA/VILI and cryotherapy were evaluated in two of the studies (*n* = 2, 8.0%).

### Efficacy and accuracy of cervical cancer screening methods in HIV-positive women

#### VIA

A number of studies [21, 22, 25, 27–30, 32, 34, 35, 43] have all reported VIA performance in detecting cervical intraepithelial neoplasia grade 2+ (CIN2+) that is generally consistent, with sensitivity of 55.0–80.0% and specificity of 65.0–83.0% (see Table 2). However, some evidence [25, 32] reported specificity of 47.3% and 51.0%, which are lower than what was found in other studies; whilst in Zambia [43], there was a reported specificity of 92.0%, which was higher than in other areas. As a diagnostic test, VIA had positive and negative predictive values of 38.6% (95% CI = 28.8–49.3%) and 79.1% (95% CI = 67.8–87.2%), respectively [32], and this was comparable to the reported positive predictive value of 35.2% [22].

**Table 2:** Clinical performance of cervical cancer screening methods/tools for detecting CIN2+

Author, year of publication	Screening method/tool	HIV-infected				HIV-negative			
		Sensitivity	Specificity	PPV	NPV	Sensitivity	Specificity	PPV	NPV
Bansil et al., 2015	VIA	77.1 (59.9–89.6)	47.3 (40.8–53.8)	17.8 (12.0–24.8)	93.3 (87.3–97.1)	93.8 (69.8–99.8)	60.5 (57.3–63.7)	3.9 (2.2–6.4)	99.8(99.0–100.0)
Chung et al., 2013	VIA	62.7 (53.4–71.2)	65.9 (60.7–70.7)	37.1 (30.5–44.2)	84.6 (79.8–88.5)				
Dartell et al., 2014	VIA	50.0 (31.5–68.5)	90.5 (87.2–93.8)	32.6	95.2	22.9 (14.5–31.3)	97.2 (96.7–97.8)	21.6	97.5
Huchko et al., 2015	VIA	84.0 (64.0–95.5)	78.6 (73.5–83.1)	24.7 (16.0–35.3)	98.3 (95.8–99.5)				
Joshi et al., 2013	VIA	83.6 (71.2–92.2)	88.8 (86.7–90.6)	27.7 (21.1–35.2)	99.1 (98.2–99.6)				
Kuhn et al., 2010	VIA	63.9 (46.2–79.2)	73.5 (67.4–78.8)	27.5 (17.8–37.3)	90.9 (85.8–96.0)	47.8 (35.7–60.2)	80.3 (78.2–82.2)	9.6 (6.5–12.7)	96.7 (95.6–97.8)
Mabeya et al., 2012	VIA	69.6	51.0	38.6	79.1				
Sahasrabuddhe et al., 2012	VIA	80.0 (66.3–90.0)	82.6 (77.4–87.1)	47.6 (36.6–58.9)	95.4 (91.8–97.8)				
Akinwuntan et al., 2008	VIA	76.0 (52.0–91.0)	83.0 (77.0–88.0)	34.0 (21.0–49.0)	97.0 (92.0–99.0)				
Firnhaber et al., 2016	VIA	65.4 (59.7–71.1)	68.5 (65.3–71.7)						
Chibwesha et al., 2016	VIA	48.0 (30.0–67.0)	92.0 (86.0–95.0)	52.0 (33.0–71.0)	91.0 (85.0–95.0)				
Huchko et al., 2015	VILI	84.2 (68.7–94.0)	76.4 (71.2–81.3)	31.7 (22.8–41.7)	97.4 (94.4–99.0)				
Joshi et al., 2013	VILI	89.1 (77.8–95.9)	89.3 (87.3–91.1)	30.1 (23.1–37.7)	99.4 (98.6–99.8)				
Chibwesha et al., 2016	DC	59.0 (41.0–76.0)	88.0 (82.0–93.0)	49.0 (32.0–65.0)	92.0 (87.0–96.0)				
Bateman et al., 2014	DC	84.0 (72.0–91.0)	58.0 (52.0–64.0)	33.0 (26.0–41.0)	93.0 (88.0–96.0)				
Joshi et al., 2013	Pap smear	63.3 (48.3–76.6)	94.5 (92.9–95.8)	35.2 (25.3–46.1)	98.2 (97.2–98.9)				
Chung et al., 2013	Pap smear	71.8 (62.8–79.4)	97.1 (94.7–98.4)	88.8 (80.5–93.8)	91.5 (88.2–93.9)				
Mabeya et al., 2013	Pap smear	52.5	66.3	39.7	76.8				
Sahasrabuddhe et al., 2012	Pap smear	60.5 (44.4–75.0)	64.6 (57.9–70.8)	24.8 (16.9–34.1)	89.4 (83.6–93.7)				
Akinwuntan et al., 2008	Pap smear	57.0 (34.0–77.0)	95.0 (90.0–97.0)	55.0 (33.0–75.0)	95.0 (91.0–98.0)				
Firnhaber et al., 2013	Pap smear	75.8 (70.8–80.8)	83.4 (80.9–85.9)						
Bateman et al., 2014	Pap smear	61.0 (48.0–72.0)	58.0 (52.0–64.0)	27.0 (20.0–35.0)	86.0 (79.0–90.0)				
Michelow et al., 2016	Cellslide® automated liquid-based cytology	76.0 (64.8–85.1)	91.0 (87.0–94.2)	70.4 (59.2–80.0)	93.1 (89.4–95.9)				
Bansil et al., 2015	Vaginal careHPV	80.0 (63.1–91.6)	59.9 (53.4–66.2)	22.8 (15.7–31.2)	95.3 (90.6–98.1)	75.0 (47.6–92.7)	81.9 (79.3–84.4)	6.7 (3.5–11.4)	99.5 (98.7–99.9)
Bansil et al., 2015	Cervical careHPV	94.3 (80.8–99.3)	62.4 (55.9–68.6)	27.0 (19.4–35.8)	98.7 (95.3–99.8)	81.3 (54.4–96.0)	80.9 (78.2–83.3)	6.8 (3.7–11.4)	99.6 (98.8–99.9)
Obiri-Yeboah et al., 2017	CareHPV	87.5 (47.3–99.7)	52.1 (44.7–59.5)	7.2 (3.0–14.3)	99.0 (94.5–100.0)				
Segondy et al., 2016 [36]	CareHPV	93.3 (83.8–98.2)	57.9 (54.5–61.2)						
Chung et al., 2013	HPV DNA test	83.6 (75.6–89.4)	55.7 (50.4–60.9)	37.7 (31.9–43.9)	91.4 (86.8–94.5)				
Dartell et al., 2014	HR HPV	100.0	58.2 (52.6.63.7)	17.9	100.0	92.7 (87.5–97.9)	85.3 (84.0–86.6)	17.2	99.7
Joshi et al., 2013	HC2 test	94.6 (84.9–98.9)	77.4 (74.8–79.9)	17.8 (13.6–22.6)	99.6 (99.0–99.9)				
Ngou et al., 2015	HC2	88.8	55.2	24.7	96.7				
Ngou et al., 2015	INNO-LiPA	92.5	35.1	19.1	96.6				
Obiri-Yeboah et al., 2017	Anyplex II HPV 28	87.5 (47.3–99.7)	38.8 (31.8–46.2)	5.7 (2.3–11.5)	98.6 (92.7–100.0)				
Firnhaber et al., 2016	HC2	91.9 (88.5–95.3)	51.4 (48.0–54.8)						
Chibwesha et al., 2016	Xpert HPV	88.0 (71.0–97.0)	60.0 (52.0–86.0)	30.0 (21.0–40.0)	96.0 (90.0–99.0)				
Chibwesha et al., 2016	OncoE6	31.0 (16.0–50.0)	99.0 (97.0–100)	91.0 (59–100)	88.0 (83.0–93.0)				
Segondy et al., 2016	INNO-LiPA	96.7 (88.5–99.6)	32.0 (29.0–35.2)						
Kuhn et al., 2010	HPV DNA	94.4 (81.3–99.3)	64.4 (58.0–70.3)	29.9 (21.3–38.6)	97.2 (87.0–99.4)	87.0 (76.7–93.9)	87.0 (85.2–88.6)	22.7(17.6–27.9)	99.0 (97.9–99.5)
Wu et al., 2016	P16INK4a cutoff level = 9 U/mL (%)	89.0	22.9	13.6	93.8				
Chung et al., 2013	VIA + HPV test	58.2 (48.8–67.0)	83.7 (79.4–87.2)	53.3 (44.4–62.0)	86.2 (82.1–89.5)				
Chung et al., 2013	VIA + Pap smear	50.9 (41.7–60.1)	99.1 (97.5–99.7)	94.9 (86.1–98.3)	86.3 (82.5–89.3)				
Chung et al., 2013	HPV + Pap smear	62.7 (53.4–71.2)	98.5 (96.6–99.4)	93.2 (85.1–97.1)	89.2 (85.7–91.9)				
Joshi et al., 2013	VIA and VILI	81.8 (69.1–90.9)	93.2 (91.5–94.6)						
Joshi et al., 2013	HC2 and VIA	80.0 (67.0–89.6)	96.0 (94.6–97.1)						
Joshi et al., 2013	HC2 and VILI	83.6 (71.2–92.2)	96.9 (95.7–97.9)						
Joshi et al., 2013	HC2 and VIA/VILI	85.5 (73.3–93.5)	95.3 (93.9–96.5)						
Joshi et al., 2013	VIA and cytology	57.1 (42.2–71.2)	98.8 (98.0–99.4)						
Joshi et al., 2013	VILI and cytology	55.1 (40.2–69.3)	99.6 (99.0–99.9)						
Joshi et al., 2013	HC2 and cytology	63.3 (48.3–76.6)	96.6 (95.3–97.6)						

*VIA* visual inspection with acetic acid, *VILI* visual inspection with Lugol’s iodine, *DC* digital cervicography, *HR-HPV* high-risk human papillomavirus, *HC2* Hybrid Capture-2, *PPV* positive predictive value, *NPV* negative predictive value

#### VILI

Using the CIN2+ threshold (see Table 2), VILI has a better sensitivity and specificity when compared to VIA, with sensitivity ranging from 68.0 to 96% and specificity of 71.0 to 91.0% [22, 29].

### Digital cervicography

Two studies in Zambia reported different efficacy of digital cervicography (DC) in screening for CIN2+ amongst HIV-positive women. The first study [43] reported a sensitivity of 59.0% (95% CI 41.0–76.0), specificity of 88.0% (82.0–93.0), positive predictive value (PPV) of 49.0% (32.0–65.0) and negative predictive value (NPV) of 92.0% (95% CI 87.0–96.0), whilst the second study [37] indicated that DC had high sensitivity of 84.0% (95% CI 72.0–91.0) but low specificity of 58.0% (95% CI 52.0–64.0), PPV of 33.0% (95% CI 26.0–41.0) and NPV of 93.0% (95% CI 88.0–96.0).

### Cytology-based tests

Sensitivity and specificity of Pap smear in detecting CIN2+ in HIV-seropositive women have been shown to be between 45.0 and 76.0% and 58.0 and 98.0%, respectively [27, 30, 32, 34, 35, 37]. This clinical performance of Pap smear was similar to Cellslide® automated liquid-based cytology which recorded a sensitivity of 76.0% (95% CI 64.8–85.1) and a specificity of 91.0% (95% CI 87.0–94.2) [33].

### Tests/tools for high-risk HPV DNA detection

The sensitivity of high-risk human papillomavirus (HR-HPV) DNA detection tests/tools such as careHPV®, HC2® test, INNO-LiPA®, Xpert HPV® and P16INK4a® is better when compared to cytology-based tests and visual tests as indicated by a sensitivity of 80.0–97%. However, the specificity of these HPV tests is similar in some cases but mostly lower to cytology or visual tests, 51.0–78.0% [21, 23–25, 27–30, 40, 42, 43]. Although the OncoE6® had a specificity of 99.0% (95% CI 97.0–100), it had a low sensitivity of between 16.0 and 50.0% [43].

### Clinical performance of combined screening methods/tests

#### VIA and Pap smear and VILI and Pap smear

Sequential testing of HIV-seropositive women with VIA and Pap smear did not result in any significant changes in sensitivity which was 50.0–72.0%, but there was a significant change in specificity (97.0–99.5%) when compared to individual VIA or Pap smear screening [27, 30]. The clinical performance of testing with both VILI and Pap smear was almost similar to using VIA and Pap smear, with sensitivity being 55.1% (95% CI 40.2–69.3%) and a slightly increased specificity of 99.6% (95% CI 99.0–99.9%).

#### VIA and HPV testing and VIA/VILI and HC2®

Some findings indicated that a combination of VIA and testing for HPV did not improve sensitivity or specificity when compared to the use of individual tests, with sensitivity of clinical performance of the combination being 58.2% (95% CI 48.8–67.0%) and specificity of 83.7% (95% CI 79.4–87.2%) [27]. However, in India, it was reported that the use of either VIA or VILI and HPV testing using HC2® showed slightly better performance with a sensitivity of 85.5% (95% CI 73.3–93.5%) and specificity of 95.3% (93.9–96.5%) [30].

#### VIA and VILI

The use of a combination of VIA and VILI in detecting CIN2+ in HIV-seropositive women resulted in an increased clinical performance with a sensitivity of 81.8% (95% CI 69.1–90.9%) and specificity of 93.2% (95% CI 91.5–94.6%) [30]. These results indicate that a combined use of both VIA and VILI can counter false-positive results that are prone when both are used as sole methods [38].

### Screen-and-treat method

In a follow-up of 36 months, screen-and-treat using HPV DNA testing and cryotherapy significantly reduced CIN2+ in HIV-positive women, with a relative risk of 0.20 (95% CI 0.06–0.69). Screen-and-treat using HPV DNA testing and cryotherapy had better positive outcomes when compared to screen-and-treat using VIA and cryotherapy [21]. In Uganda, the findings indicated that using VIA and cryotherapy alone has the potential of resulting in overtreatment of patients because of high false-positive rates [38]. To reduce these high false-positive complications, a see-see and treat method using VIA, colposcopy and cryotherapy was seen to be effective as it reduced overtreatment by 72% (439/625) [38].

### Quality assessment of included studies

Overall, most of the studies (*n* = 16, 64.0%) were determined to be of moderate quality, that is, a score of 3 ‘yes’ out of 5 on the quality scale. Only four studies (16.0%) were considered ‘high’ quality, that is, a score of 4 ‘yes’ out of 5. Five studies (20.0%) were considered to be of low quality and had a score of 2 ‘yes’ or below out of 5.

Only five studies (20.0%) had control groups, and this made it difficult to confidently ascertain if the reported findings were due to the screening method or it was by chance. Most of the studies did not evaluate the value of the screening modalities since they did not follow up the screened individuals to fully assess their effectiveness or account for disease regression or progression. Although a few studies followed up the screened HIV-positive women, the follow-up period was not adequate to measure the effectiveness of the screening methods or offer reasons for lost to follow-up of those who were due for their second screening procedure. Some of the studies did not measure confounders or include them in their analyses, with a few mentioning confounders and their expected influence on the results.

There were limited study design and methodology description in some articles, and these made it difficult to gauge if the reported findings were from an evaluative programme instead of a rigorous research. There was no mention of how participants were randomised or if randomisation was conducted in some of the studies that had a control group, and this made it difficult to attribute the reported results to the evaluated screening methods. In studies that evaluated a number of screening methods, there is a likelihood that some might have overestimated the sensitivity and specificity of the screening methods because in their analyses, they failed to calculate a dichotomous result to cater for those with negative screening results from other methods.

## Discussion

The high-risk rate that HIV-seropositive women have towards developing cervical cancer renders the lack of specific evidence on which cervical cancer screening method is suitable and effective for them, a public health challenge. This review attempts to offer evidence on which cervical cancer screening approach or method is ‘better’ for HIV-seropositive women in developing countries and offer policymakers and health leadership a base to formulate solid screening guidelines.

This review has shown that there is not yet a standard screening method/tool for cervical cancer screening amongst HIV-seropositive women because each method has its benefits and risks that require to be considered when using it. However, this risk-benefit scale is usually considered secondary in developing countries because the availability of a screening method, whether effective or not, is important. In addition, this review has shown that there is no better screening method that fits the healthcare system of every developing country because priorities, resources and implementation of guidelines are different. Since all the cervical cancer screening methods being used for HIV-positive women are the same for HIV-negative women, careful analysis of each method’s risks and benefits is required to help decisions on which method to use in the meantime as further research is conducted to find the ‘best’ screening method.

**Fig. 3:**
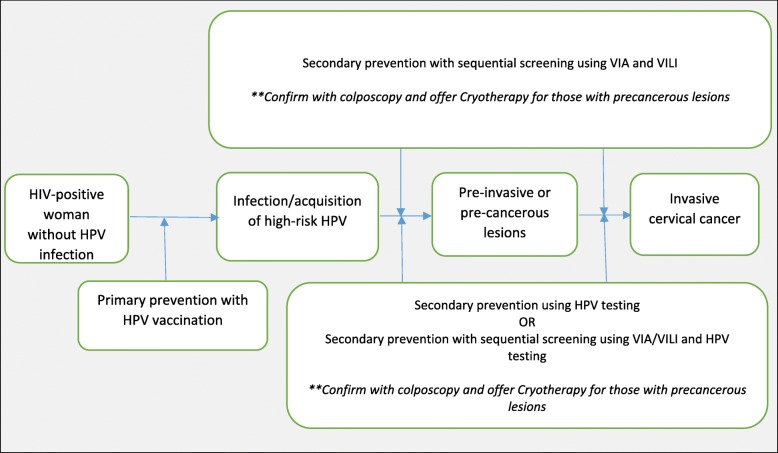
Proposed opportunities for prevention of cervical cancer in HIV-seropositive women. **Treatment with cryotherapy or LEEP should be offered after the results are verified by qualified personnel to limit subjecting these patients to unnecessary treatments. HPV, human papillomavirus; VIA, visual inspection with acetic acid; VILI, visual inspection with Lugol’s iodine; LEEP, loop electrosurgical excision procedure

Due to the challenges in establishing Pap smear as a national screening programme as has been done in developed countries, the use of VIA as the screening method of choice amongst both HIV-positive and HIV-negative women has increased significantly in developing countries [14, 45]. In as much as VIA is being used more often because of its easy applicability even by nurses, evidence have shown that the use of VILI can increase the efficacy and accuracy amongst HIV-positive women [20, 30]. The performance of VIA was reported to be much better in HIV-negative than HIV-positive women [25], and there are more high false-positive rates amongst HIV-positive women [34, 38]. These findings might indicate that sequential screening using both VIA and VILI may be beneficial amongst HIV-seropositive women (see proposed opportunities in Fig. 3). The sequential use of VIA and VILI has indicated a better clinical performance and risk-benefit balance when compared to their use individually [20], and this might be a combination method that developing countries can use for HIV-seropositive women. However, VILI’s use in developing countries is not at the same scale as that of VIA because of the cost issues associated with iodine when compared to acetic acid. In addition, lessons learnt from Ethiopia indicated that implementation of visual-based screening methods amongst HIV-seropositive women requires provider initiation as a complimenting element [46].

As the implementation of VIA and VILI continue to grow in developing countries, the risks of misdiagnosing associated with visual inspection methods (VIA and VILI) should be carefully monitored amongst HIV-seropositive to prevent subjecting these women to unnecessary treatment as well as waste resources. This is supported by synthesised evidence from a review of the visual inspection methods [45]. Therefore, developing countries may be better off using VIA and VILI as screening tools not as diagnostic tests. In addition, the see-see-and treat combination using VIA or VILI coupled with colposcopy and treating with cryotherapy has the potential of significantly reducing false positives and preventing overtreating in clients who will not need cryotherapy [38].

Detecting of HR-HPV has been shown to be an effective secondary screening method for cervical cancer amongst HIV-seropositive, with almost all the HPV tests indicating better clinical performance when compared to cytology-based and visual-based tests [23–25, 27, 28, 30, 36, 39, 40]. With long-term persistent infection with HPV almost always associated with the development of cervical cancer [47–50], emerging evidence suggest HPV testing as a better way as compared to cytology-based or visual screening methods [51]. A sequential screening of cervical cancer using VIA or VILI and HPV testing maybe ideal in developing countries as this will reduce the number of false-positive results [30] and hence might limit the resources and prevent subjecting women to unnecessary treatments (see Fig. 3). This combination of VIA or VILI and HPV testing has the potential to offer a better benefit-risk balance when compared to other available screening methods currently being used for HIV-seropositive women. However, for developing countries to implement such change, resources, guidelines and policies (which are context-specific) will need to be made available in line with emerging scientific evidence. In addition, the safety interpretation of results of HPV tests requires trained professional to limit overestimation of precancerous lesions in HIV-seropositive women, which may result in unnecessarily subjecting women to treatment that they do not need as well as waste the limited resources. Therefore, this requires training of healthcare workers when implementing HPV testing.

With improved knowledge and understanding of cervical cancer and HPV, several studies [52–57] have indicated the immunogenicity and safety of the currently used HPV vaccine amongst young and middle-aged HIV-seropositive women. There is scanty data on implementation of the HPV vaccination, its efficacy and uptake amongst HIV-positive women as the available evidence focuses on the safety and immunogenicity [52–58]. However, guidelines by the Centers for Disease Control and Prevention and HIV Medicine Association of Infectious Diseases Society of America recommend HPV vaccination amongst young HIV-seropositive women aged 13 to 26 years [59]. Most developing countries have embarked on mass HPV vaccination of young girls, and opportunities for effective and sustainable implementation of the vaccine amongst HIV-positive young girls exist and should be utilised. As the implementation of the mass HPV vaccination intensifies in developing countries, opportunities to increase the age of recipients to include middle-aged HIV-seropositive women should be explored and initiated as suggested in Fig. 3.

### Analytic frameworks for decision-making in screening

For developing countries, questions on how to implement and sustain cervical cancer screening in light of limited resources (human and financial), inadequate infrastructure and lack of screening programmes still exist. The questions continue to have an impact on decision-making towards screening and even prioritisation of HIV-seropositive women. In as much as this review has generated synthesised evidence on cervical cancer screening of HIV-seropositive women, utilisation and implementation of some of this evidence will be context-specific. A number of analytic frameworks for decision-making in cervical cancer screening exist [60–63], and these frameworks may help developing countries in identifying cost-effective strategies towards screening of HIV-seropositive women. These analytic frameworks can assist countries to make decisions after considering the provided evidence, epidemiological factors, political and economic factors and issues around equity and costumers’ preferences [64]. Such a transparent and systematic way of making decisions has been shown to have a positive impact on screening [61, 63].

### Limitations

The overall quality of evidence of the included studies, which was ‘moderate’, made it difficult to draw emphatic conclusions on which screening method/tool is effective on HIV-seropositive women and which one is suitable for low-income countries. The validity of the results was decreased by the risk of bias associated with the study designs, completeness of data and lack of explanations on the statistical analyses conducted. Lastly, by limiting the searching to studies reported in English, this review might have missed some relevant studies published in other languages.

## Conclusion

HIV-seropositive women are a high-risk group for developing cancer [5, 7], and identifying the ideal cervical cancer screening method for them will go a long way in reducing premature mortality amongst them. Findings of this review indicate a need for further research, mostly randomised controlled trials, that allows adequate follow-up of screened HIV-seropositive women and provide evidence on which screening method is best to use, taking into account age, one visit vs. return visit schemes, primary screening then triage, opportunistic vs. organised screening, CD4+ counts, antiretroviral therapy and quality of life.

Sequential screening using HPV test and VIA or VILI has the potential to offer a better catch of at-risk HIV-positive women [30] when compared to the other available screening methods, and this can be a solid foundation that developing countries can start to formulate their cervical cancer screening guidelines for HIV-seropositive women. However, as indicated before, there is a need for further research that will provide evidence on the best way of using this combination since it was reported that such sequential screening did not improve sensitivity or specificity [27].

Secondly, with the introduction of mass HPV vaccination amongst school-going young girls, there exist potential opportunities to offer the vaccine to middle-aged HIV-seropositive women in developing countries within well-established HIV programmes. With HPV vaccine offering more than 12 months protection in HIV-seropositive women [15], this might be a cost-effective and simple method to offer cervical cancer prevention amongst these women. In addition, HPV vaccination will offer a solution to the lack of adequate suitable infrastructure and trained professionals that has hampered Pap smear screening in developing countries.

Developing countries should strive to offer both opportunistic and organised coordinated screening programmes in the form of provider initiated. Furthermore, there is a need to expand the integration of provider-initiated cervical cancer screening services in already existing HIV services so as to enable early detection and treatment and offer a ‘one-stop’ shop. Developing countries can think of individualising cervical cancer screening depending on their available resources and context to cater for the benefit/risk of different screening methods and the general health status of the HIV-seropositive women. This is in light with the proposed see-see and treat method where the potential of high false positives and over treating can be reduced significantly [38].

### Implications of the review’s results in evidence-based health care

Based on the proposed opportunities for prevention of cervical cancer in HIV-seropositive women (see Fig. 3), a number of key messages around the reliability of the found evidence are beginning to emerge.

There is no best available cervical cancer screening method/tool for HIV-seropositive women, and the current presented evidence can be effective in certain contexts but not all. Future research can explore the feasibility, appropriateness, meaningfulness and cost-effectiveness of HPV vaccine; use of the see-see and treat using VIA/VILI, colposcopy and cryotherapy; and sequential screening using VIA/VILI and or HPV testing in HIV-burdened countries. Whatever method is to be used, the invention of a systematic screening approach, which could be helpful, should be investigated and based on cervical cancer analytic frameworks to allow transparent and systematic decision-making [61, 64].

Clients or patients should at least have an option to decide on which screening method they would prefer based on the risk-benefit balance, and this should be guided by professional judgement of healthcare staff.

## Additional files

Additional file 1: PubMed and OvidSP (MEDLINE and Embase) search strategies. This file contains two examples of the search strategies used to search for studies that were included in this literature review. The search strategies are for PubMed, MEDLINE and Embase databases. (PDF 12 kb)

Additional file 2: List of excluded studies. This file contains a list of the studies that were excluded at the full-text reading stage during the screening process. (PDF 43 kb)

## Abbreviations

CIN2+: Cervical intraepithelial neoplasia grade 2+
CINAHL: Cumulative Index to Nursing and Allied Health Literature
DC: Digital cervicography
EMBASE: Excerpta Medica Database
HC2: Hybrid Capture-2
HIV: Human immunodeficiency virus
HPV DNA/mRNA: Human papillomavirus deoxyribonucleic acid/messenger RNA
HPV: Human papillomavirus
PRISMA: Preferred Reporting Items for Systematic Review and Meta-Analysis
RCT: Randomised controlled trial
VIA: Visual inspection with acetic acid
VILI: Visual inspection with Lugol’s iodine
