# Supplementary material for: Prevention of cervical cancer in HIV-seropositive women from developing countries through cervical cancer screening: a systematic review
Source: Syst Rev. 2018 Nov 17;7:198. doi: 10.1186/s13643-018-0874-7 (PMC6240280; doi:10.1186/s13643-018-0874-7)
Supplement: Supplementary file 2 — List of excluded studies. This file contains a list of the studies that were excluded at the full-text reading stage during the screening process. (PDF 43 kb) [file 13643_2018_874_MOESM2_ESM.pdf]

File name: Additional file 2
Title of data: List of excluded studies

Adibe, M. O., & Aluh, D. O. (2017). Awareness, Knowledge and Attitudes Towards Cervical Cancer Amongst HIV-Positive Women Receiving Care in a Tertiary Hospital in Nigeria. *J Cancer Educ*.

Akarolo-Anthony, S. N., Al-Mujtaba, M., Famooto, A. O., Dareng, E. O., Olaniyan, O. B., Offiong, R., et al. (2013). HIV associated high-risk HPV infection among Nigerian women. *BMC Infect Dis*, *13*, 521.

Ali-Risasi, C., Verdonck, K., Padalko, E., Vanden Broeck, D., & Praet, M. (2015). Prevalence and risk factors for cancer of the uterine cervix among women living in Kinshasa, the Democratic Republic of the Congo: a cross-sectional study. *Infect Agent Cancer, 10*, 20.

Anastos, K., Hoover, D. R., Burk, R. D., Cajigas, A., Shi, Q., Singh, D. K., et al. (2010). Risk factors for cervical precancer and cancer in HIV-infected, HPV-positive Rwandan women. *PLoS One, 5*(10), e13525.

Baay, M. F., Kjetland, E. F., Ndhlovu, P. D., Deschoolmeester, V., Mduluza, T., Gomo, E., et al. (2004). Human papillomavirus in a rural community in Zimbabwe: the impact of HIV co-infection on HPV genotype distribution. *J Med Virol, 73*(3), 481-485.

Bailey, H., Thorne, C., Semenenko, I., Malyuta, R., Tereschenko, R., Adeyanova, I., et al. (2012). Cervical screening within HIV care: findings from an HIV-positive cohort in Ukraine. *PLoS One, 7*(4), e34706.

Banura, C., Mirembe, F. M., Katahoire, A. R., Namujju, P. B., Mbonye, A. K., & Wabwire, F. M. (2011). Epidemiology of HPV genotypes in Uganda and the role of the current preventive vaccines: A systematic review. *Infect Agent Cancer, 6*(1), 11.

Baranoski, A. S., Horsburgh, C. R., Cupples, L. A., Aschengrau, A., & Stier, E. A. (2011). Risk Factors for Nonadherence with Pap Testing in HIV-Infected Women. *Journal of Women's Health (15409996), 20*(11), 1635-1643.

Bateman, A. C., Chibwesha, C. J., & Parham, G. P. (2015). Minimizing verification bias in cervical cancer screening of HIV-infected women. *Int J Gynaecol Obstet, 128*(3), 269-270.

Bayu, H., Berhe, Y., Mulat, A., & Alemu, A. (2016). Cervical Cancer Screening Service Uptake and Associated Factors among Age Eligible Women in Mekelle Zone, Northern Ethiopia, 2015: A Community Based Study Using Health Belief Model. *PLoS One, 11*(3), e0149908.

Belete, N., Tsige, Y., & Mellie, H. (2015). Willingness and acceptability of cervical cancer screening among women living with HIV/AIDS in Addis Ababa, Ethiopia: a cross

sectional study. *Gynecol Oncol Res Pract, 2*, 6.

Blitz, S., Baxter, J., Raboud, J., Walmsley, S., Rachlis, A., Smaill, F., et al. (2013). Evaluation of HIV and Highly Active Antiretroviral Therapy on the Natural History of Human Papillomavirus Infection and Cervical Cytopathologic Findings in HIV-Positive and High-Risk HIV-Negative Women. *Journal of Infectious Diseases, 208*(3), 454-462.

Blumenthal, P. D., Gaffikin, L., Deganus, S., Lewis, R., Emerson, M., & Adadevoh, S. (2007). Cervical cancer prevention: safety, acceptability, and feasibility of a single-visit approach in Accra, Ghana. *American Journal of Obstetrics & Gynecology, 196*(4), 407-409.

Boncz, I., Suba, E. J., Geisinger, K. R., Zarka, M. A., Raab, S. S., Wright, T. C., Jr., et al. (2006). Prevention of cervical cancer in low-resource settings...Denny L, Kuhn L, De Souza M et al. Screen-and-treat approaches for cervical cancer prevention in low-resource settings: a randomized controlled trial. JAMA. 294;2173-2181. *JAMA: Journal of the American Medical Association, 295*(11), 1248-1249.

Boon, M. E., van der Veen, G., Barlow, Y., Graudenz, M. S., & Kok, L. P. (2001). Presence of proliferating (MiB-1-positive) cells in cervical smears of women infected with HIV is associated with clinical outcome: a study of Brazilian women. *Diagn Cytopathol, 24*(6), 373-377.

Bowa, K., Malyangu, E., Wood, C., & Angelleti, P. (2010). A pilot study to compare HIV status and cervical and penile pathology among couples attending the urology unit at the University Teaching Hospital Lusaka, Zambia. *Med J Zambia, 37*(2), 84-88.

Bradford, L., & Goodman, A. (2013). Cervical Cancer Screening and Prevention in Low-resource Settings. *Clinical Obstetrics & Gynecology, 56*(1), 76-87.

Carr, K. C., & Sellors, J. W. (2004). Cervical cancer screening in low resource settings using visual inspection with acetic acid. *Journal of Midwifery & Women's Health, 49*(4), 329-337.

Catarino, R., Vassilakos, P., Tebeu, P. M., Schafer, S., Bongoe, A., & Petignat, P. (2016). Risk factors associated with human papillomavirus prevalence and cervical neoplasia among Cameroonian women. *Cancer Epidemiol, 40*, 60-66.

Chama, C. M., Nggada, H., & Gashau, W. (2005). Cervical dysplasia in HIV infected women in Maiduguri, Nigeria. *J Obstet Gynaecol, 25*(3), 286-288.

Chambuso, R. S., Shadrack, S., Lidenge, S. J., Mwakibete, N., & Medeiros, R. M. (2017). Influence of HIV/AIDS on Cervical Cancer: A Retrospective Study From Tanzania. *J Glob Oncol, 3*(1), 72-78.

Chapman, C. L., & Harris, A. L. (2016). Cervical Cancer Screening for Women Living With HIV. *Nursing for Women's Health, 20*(4), 392-398.

Chapman Lambert, C. L. (2013). Factors Influencing Cervical Cancer Screening in Women Infected With HIV: A Review of the Literature. *JANAC: Journal of the Association of Nurses in AIDS Care, 24*(3), 189-197.

Chirenje, Z. M. (2005). HIV and cancer of the cervix. *Best Pract Res Clin Obstet Gynaecol, 19*(2), 269-276.

Clarke, B., & Chetty, R. (2002). Postmodern cancer: the role of human immunodeficiency virus in uterine cervical cancer. *Mol Pathol, 55*(1), 19-24.

Clifford, G. M., Goncalves, M. A., & Franceschi, S. (2006). Human papillomavirus types among women infected with HIV: a meta-analysis. *Aids, 20*(18), 2337-2344.

Coelho Lima, B. M., Golub, J. E., Tonani Mattos, A., Freitas, L. B., Cruz Spano, L., & Espinosa Miranda, A. (2009). Human papillomavirus in women with and without HIV-1 infection attending an STI clinic in Vitoria, Brazil. *J Int Assoc Physicians AIDS Care (Chic), 8*(5), 286-290.

Crosbie, E. J., Einstein, M. H., Franceschi, S., & Kitchener, H. C. (2013). Human papillomavirus and cervical cancer. *Lancet, 382 North American Edition*(9895), 889-899.

Cross, S. L., Suharwardy, S. H., Bodavula, P., Schechtman, K., Overton, E. T., Onen, N. F., et al. (2014). Improving cervical cancer screening rates in an urban HIV clinic. *AIDS Care, 26*(9), 1186-1193.

Dahiya, N., Bachani, D., Acharya, A. S., Sharma, D. N., Gupta, S., & Haresh, K. P. (2017). Socio-Demographic, Reproductive and Clinical Profile of Women Diagnosed with Advanced Cervical Cancer in a Tertiary Care Institute of Delhi. *J Obstet Gynaecol India, 67*(1), 53-60.

Dal Maso, L., Franceschi, S., Lise, M., De' Bianchi, P. S., Polesel, J., Ghinelli, F., et al. (2010). Self-reported history of Pap-smear in HIV-positive women in Northern Italy: a cross-sectional study. *BMC Cancer, 10*, 310.

Davis, M. A., Gray, R. H., Grabowski, M. K., Serwadda, D., Kigozi, G., Gravitt, P. E., et al. (2013). Male circumcision decreases high-risk human papillomavirus viral load in female partners: a randomized trial in Rakai, Uganda. *Int J Cancer, 133*(5), 1247-1252.

de Mattos, A. T., de Freitas, L. B., Lima, B. M., Miranda, A. E., & Spano, L. C. (2011). Diversity and uncommon HPV types in HIV seropositive and seronegative women attending an STI clinic. *Braz J Microbiol, 42*(2), 786-793.

De Vuyst, H., Chung, M. H., Baussano, I., Mugo, N. R., Tenet, V., van Kemenade, F. J., et al. (2013). Comparison of HPV DNA testing in cervical exfoliated cells and tissue biopsies among HIV-positive women in Kenya. *Int J Cancer, 133*(6), 1441-1446.

De Vuyst, H., Mugo, N. R., Chung, M. H., McKenzie, K. P., Nyongesa-Malava, E., Tenet, V., et al. (2012). Prevalence and determinants of human papillomavirus infection and cervical lesions in HIV-positive women in Kenya. *Br J Cancer, 107*(9), 1624-1630.

Delgado, J. R., Menacho, L., Segura, E. R., Roman, F., & Cabello, R. (2017). Cervical cancer screening practices, knowledge of screening and risk, and highly active antiretroviral therapy adherence among women living with human immunodeficiency virus in Lima, Peru. *Int J STD AIDS, 28*(3), 290-293.

Delory, T., Ngo-Giang-Huong, N., Rangdaeng, S., Chotivanich, N., Limtrakul, A., Putiyanun, C., et al. (2017). Human Papillomavirus infection and cervical lesions in HIV infected women on antiretroviral treatment in Thailand. *J Infect, 74*(5), 501-511.

Dim, C. C., Dim, N. R., Ezegwui, H. U., & Ikeme, A. C. (2009). An unmet cancer screening need of HIV-positive women in southeastern Nigeria. *Medscape J Med, 11*(1), 19.

Dim, C. C., Onyedum, C. C., Dim, N. R., & Chukwuka, J. C. (2015). Cervical Cancer Screening among HIV-Positive Women in Nigeria: An Assessment of Use and Willingness to Pay in the Absence of Donor Support. *Journal of the International Association of Providers of AIDS Care, 14*(3), 241-244.

Doutre, S., Omar, T., Goumbri-Lompo, O., Kelly, H., Clavero, O., Zan, S., et al. (2018). Cervical intraepithelial neoplasia (CIN) in African women living with HIV: role and effect of rigorous histopathological review by a panel of pathologists in the HARP study endpoint determination. *J Clin Pathol, 71*(1), 40-45.

Dube Mandishora, R. S., Christiansen, I. K., Chin'ombe, N., Duri, K., Ngara, B., Rounge, T. B., et al. (2017). Genotypic diversity of anogenital human papillomavirus in women attending cervical cancer screening in Harare, Zimbabwe. *J Med Virol, 89*(9), 1671-1677.

Ebu, N. I., & Ogah, J. K. (2018). Predictors of cervical cancer screening intention of HIV-positive women in the central region of Ghana. *BMC Womens Health, 18*(1), 43.

Ene, L., Voinea, C., Stefanescu, C., Sima, D., Duiculescu, D., & Mehta, S. R. (2016). Cervical HPV infection in Romanian women infected with HIV during early childhood. *Int J STD AIDS, 27*(12), 1079-1085.

Erku, D. A., Netere, A. K., Mersha, A. G., Abebe, S. A., Mekuria, A. B., & Belachew, S. A. (2017). Comprehensive knowledge and uptake of cervical cancer screening is low among women living with HIV/AIDS in Northwest Ethiopia. *Gynecol Oncol Res Pract, 4,* 20.

Falloon, I. R. (2000). General practice recruitment for people at risk of schizophrenia: the Buckingham experience. *The Australian and New Zealand journal of psychiatry, 34 Suppl*, S131-136; discussion S140.

Feola, T. D., Albert, M. B., Shahabi, K., & Endy, T. (2013). Prevalence of HPV in HIV-Infected Women in the Designated AIDS Center at Upstate Medical University and the Potential Benefit of Vaccination Regardless of Age. *JANAC: Journal of the Association of Nurses in AIDS Care, 24*(2), 176-179.

Firnhaber, C., Van Le, H., Pettifor, A., Schulze, D., Michelow, P., Sanne, I. M., et al. (2010). Association between cervical dysplasia and human papillomavirus in HIV seropositive women from Johannesburg South Africa. *Cancer Causes Control, 21*(3), 433-443.

Fletcher, F., Vidrine, D., Tami-Maury, I., Danysh, H., King, R., Buchberg, M., et al. (2014). Cervical Cancer Screening Adherence among HIV-Positive Female Smokers from a Comprehensive HIV Clinic. *AIDS & Behavior, 18*(3), 544-554.

Fletcher, F. E., Buchberg, M., Schover, L. R., Basen-Engquist, K., Kempf, M.-C., Arduino, R. C., et al. (2014). Perceptions of barriers and facilitators to cervical cancer screening among low-income, HIV-infected women from an integrated HIV clinic. *AIDS Care, 26*(10), 1229-1235.

Fokom-Domgue, J., Combescure, C., Fokom-Defo, V., Tebeu, P. M., Vassilakos, P., Kengne, A. P., et al. (2015). Performance of alternative strategies for primary cervical cancer screening in sub-Saharan Africa: systematic review and meta-analysis of diagnostic test accuracy studies. *Bmj, 351*, h3084.

Ford, C. (2011). Implementation of cervical cancer prevention services for HIV-infected women in Zambia: measuring program effectiveness. *Journal of Family Planning & Reproductive Health Care, 37*(3), 186-186.

Franceschi, S., & Jaffe, H. (2007). Cervical cancer screening of women living with HIV infection: a must in the era of antiretroviral therapy. *Clin Infect Dis, 45*(4), 510-513.

Frati, E. R., Martinelli, M., Fasoli, E., Colzani, D., Bianchi, S., Binda, S., et al. (2015). HPV Testing from Dried Urine Spots as a Tool for Cervical Cancer Screening in Low-Income Countries. *BioMed Research International, 2015*, 1-5.

Frazier, E. L., Sutton, M. Y., Tie, Y., McNaghten, A. D., Blair, J. M., & Skarbinski, J. (2016). Screening for Cervical Cancer and Sexually Transmitted Diseases Among HIV-Infected Women. *Journal of Women's Health (15409996), 25*(2), 124-132.

Gaikwad, N. L., Mahajan, N. N., & Mahajan, K. N. (2007). Re: alternative cervical cancer prevention in low-resource settings: experiences of visual inspection by acetic acid with single-visit approach in the first five provinces of Thailand...Aust N Z J Obstet Gynaecol. 2007 Feb;47(1):54-60. *Australian & New Zealand Journal of Obstetrics & Gynaecology, 47*(3), 258-259.

Gedefaw, A., Astatkie, A., & Tessema, G. A. (2013). The prevalence of precancerous cervical cancer lesion among HIV-infected women in southern Ethiopia: a cross-sectional study.

PLoS One, 8(12), e84519.

Gizaw, M., Addissie, A., Getachew, S., Ayele, W., Mitiku, I., Moelle, U., et al. (2017). Cervical cancer patients presentation and survival in the only oncology referral hospital, Ethiopia: a retrospective cohort study. *Infect Agent Cancer, 12,* 61.

Goldie, S. J., Weinstein, M. C., Kuntz, K. M., & Freedberg, K. A. (1999). The costs, clinical benefits, and cost-effectiveness of screening for cervical cancer in HIV-infected women. *Annals of Internal Medicine, 130*(2), 97-107.

Hanisch, R. A., Cherne, S. L., Sow, P. S., Winer, R. L., Hughes, J. P., Feng, Q., et al. (2014). Human papillomavirus type 16 viral load in relation to HIV infection, cervical neoplasia and cancer in Senegal. *Cancer Epidemiol, 38*(4), 369-375.

Hanisch, R. A., Sow, P. S., Toure, M., Dem, A., Dembele, B., Toure, P., et al. (2013). Influence of HIV-1 and/or HIV-2 infection and CD4 count on cervical HPV DNA detection in women from Senegal, West Africa. *J Clin Virol, 58*(4), 696-702.

Hank, E., Hoque, M. E., & Zungu, L. (2013). Cervical precancerous lesions and cancer among patients in the gynaecology outpatient department at a tertiary hospital in South Africa. *Asian Pac J Cancer Prev, 14*(8), 4903-4906.

Harris, T. G., Burk, R. D., Palefsky, J. M., Massad, L. S., Bang, J. Y., Anastos, K., et al. (2005). Incidence of cervical squamous intraepithelial lesions associated with HIV serostatus, CD4 cell counts, and human papillomavirus test results. *JAMA: Journal of the American Medical Association, 293*(12), 1471-1476.

Hawes, S. E., Critchlow, C. W., Sow, P. S., Toure, P., N'Doye, I., Diop, A., et al. (2006). Incident high-grade squamous intraepithelial lesions in Senegalese women with and without human immunodeficiency virus type 1 (HIV-1) and HIV-2. *J Natl Cancer Inst, 98*(2), 100-109.

Izudi, J., Adrawa, N., & Amongin, D. (2016). Precancerous Cervix in Human Immunodeficiency Virus Infected Women Thirty Years Old and above in Northern Uganda. *J Oncol, 2016,* 5473681.

Jaquet, A., Horo, A., Charbonneau, V., Ekouevi, D. K., Roncin, L., Toure, B., et al. (2012). Cervical human papillomavirus and HIV infection in women of child-bearing age in Abidjan, Cote d'Ivoire, 2010. *Br J Cancer, 107*(3), 556-563.

Joshi, S., Babu, J. M., Jayalakshmi, D., Kulkarni, V., Divate, U., Muwonge, R., et al. (2014). Human papillomavirus infection among human immunodeficiency virus-infected women in Maharashtra, India. *Vaccine, 32*(9), 1079-1085.

Kadhel, P., Multigner, L., Bardinet, F., Goerger-Sow, M., & Janky, E. (2012). Cervical intraepithelial neoplasia and invasive cancer risks in women infected with HIV in the

French West Indies. *HIV Med, 13*(1), 79-82.

Kafuruki, L., Rambau, P. F., Massinde, A., & Masalu, N. (2013). Prevalence and predictors of Cervical Intraepithelial Neoplasia among HIV infected women at Bugando Medical Centre, Mwanza-Tanzania. *Infect Agent Cancer, 8*(1), 45.

Kahesa, C., Kjaer, S. K., Ngoma, T., Mwaiselage, J., Dartell, M., Iftner, T., et al. (2012). Risk factors for VIA positivity and determinants of screening attendances in Dar es Salaam, Tanzania. *BMC Public Health, 12*, 1055.

Kangmennaang, J., Onyango, E. O., Luginaah, I., & Elliott, S. J. (2018). The next Sub Saharan African epidemic? A case study of the determinants of cervical cancer knowledge and screening in Kenya. *Soc Sci Med, 197*, 203-212.

Kohler, R. E., Tang, J., Gopal, S., Chinula, L., Hosseinipour, M. C., Liomba, N. G., et al. (2016). High rates of cervical cancer among HIV-infected women at a referral hospital in Malawi. *Int J STD AIDS, 27*(9), 753-760.

Kreiss, J. K., Kiviat, N. B., Plummer, F. A., Roberts, P. L., Waiyaki, P., Ngugi, E., et al. (1992). Human immunodeficiency virus, human papillomavirus, and cervical intraepithelial neoplasia in Nairobi prostitutes. *Sex Transm Dis, 19*(1), 54-59.

Kronenberg, C., Doran, T., Goddard, M., Kendrick, T., Gilbody, S., Dare, C. R., et al. (2017). Identifying primary care quality indicators for people with serious mental illness: a systematic review. *The British journal of general practice : the journal of the Royal College of General Practitioners, 67*(661), e519-e530.

Kumakech, E., Andersson, S., Wabinga, H., & Berggren, V. (2015). Integration of HIV and cervical cancer screening perceptions and preferences of communities in Uganda. *BMC Womens Health, 15*, 23.

Kumakech, E., Andersson, S., Wabinga, H., Musubika, C., Kirimunda, S., & Berggren, V. (2017). Cervical cancer risk perceptions, sexual risk behaviors and sexually transmitted infections among Bivalent Human Papillomavirus vaccinated and non-vaccinated young women in Uganda - 5 year follow up study. *BMC Womens Health, 17*(1), 40.

LaRusso, L. (2012). Cervical Cancer Screening in Women with HIV. *Nursing for Women's Health, 16*(5), 363-368.

Leece, P., Kendall, C., Touchie, C., Pottie, K., Angel, J. B., & Jaffey, J. (2010). Cervical cancer screening among HIV-positive women. Retrospective cohort study from a tertiary care HIV clinic. *Canadian Family Physician, 56*(12), e425-431.

Leon-Maldonado, L., Wentzell, E., Brown, B., Allen-Leigh, B., Torres-Ibarra, L., Salmeron, J., et al. (2016). Perceptions and Experiences of Human Papillomavirus (HPV) Infection and Testing among Low-Income Mexican Women. *PLoS One, 11*(5), e0153367.

Leroy, V., Ladner, J., De Clercq, A., Meheus, A., Nyiraziraje, M., Karita, E., et al. (1999). Cervical dysplasia and HIV type 1 infection in African pregnant women: a cross sectional study, Kigali, Rwanda. The Pregnancy and HIV Study Group (EGE). *Sex Transm Infect, 75*(2), 103-106.

Leslie, H. H., Karasek, D. A., Harris, L. F., Chang, E., Abdulrahim, N., Maloba, M., et al. (2014). Cervical cancer precursors and hormonal contraceptive use in HIV-positive women: application of a causal model and semi-parametric estimation methods. *PLoS One, 9*(6), e101090.

Levin, J. B., Krivenko, A., Howland, M., Schlachet, R., & Sajatovic, M. (2016). Medication Adherence in Patients with Bipolar Disorder: A Comprehensive Review. *CNS drugs, 30*(9), 819-835.

Lince-Deroche, N., Phiri, J., Michelow, P., Smith, J. S., & Firnhaber, C. (2015). Costs and Cost Effectiveness of Three Approaches for Cervical Cancer Screening among HIV-Positive Women in Johannesburg, South Africa. *PLoS One, 10*(11), e0141969.

Logan, J. L., Khambaty, M. Q., D'Souza, K. M., & Menezes, L. J. (2010). Cervical Cancer Screening Among HIV-Infected Women in a Health Department Setting. *AIDS Patient Care & STDs, 24*(8), 471-475.

Lovgren, K., Soliman, A. S., Ngoma, T., Kahesa, C., & Meza, J. (2016). Characteristics and geographic distribution of HIV-positive women diagnosed with cervical cancer in Dar es Salaam, Tanzania. *Int J STD AIDS, 27*(12), 1049-1056.

Luchters, S. M., Vanden Broeck, D., Chersich, M. F., Nel, A., Delva, W., Mandaliya, K., et al. (2010). Association of HIV infection with distribution and viral load of HPV types in Kenya: a survey with 820 female sex workers. *BMC Infect Dis, 10*, 18.

Luz, P. M., Velasque, L., Friedman, R. K., Russomano, F., Andrade, A. C., Moreira, R. I., et al. (2012). Cervical cytological abnormalities and factors associated with high-grade squamous intraepithelial lesions among HIV-infected women from Rio de Janeiro, Brazil. *Int J STD AIDS, 23*(1), 12-17.

Macleod, I. J., O'Donnell, B., Moyo, S., Lockman, S., Shapiro, R. L., Kayembe, M., et al. (2011). Prevalence of human papillomavirus genotypes and associated cervical squamous intraepithelial lesions in HIV-infected women in Botswana. *J Med Virol, 83*(10), 1689-1695.

Mahomed, K., Evans, D., Sauls, C., Richter, K., Smith, J., & Firnhaber, C. (2014). Human papillomavirus (HPV) testing on self-collected specimens: perceptions among HIV positive women attending rural and urban clinics in South Africa. *Pan Afr Med J, 17*, 189.

Malambo, N., & Erikson, S. (2017). 'Worse than HIV': The logics of cancer screening avoidance in Swaziland. *Glob Public Health*, 1-11.

Mane, A., Nirmalkar, A., Risbud, A. R., Vermund, S. H., Mehendale, S. M., & Sahasrabuddhe, V. V. (2012). HPV genotype distribution in cervical intraepithelial neoplasia among HIV-infected women in Pune, India. *PLoS One, 7*(6), e38731.
